# The social brain: allowing humans to boldly go where no other species has been

**DOI:** 10.1098/rstb.2009.0160

**Published:** 2010-01-12

**Authors:** Uta Frith, Chris Frith

**Affiliations:** 1 ICN, UCL, 17 Queen Square, London, UK; 2 Wellcome Centre for Neuroimaging, UCL, London, UK; 3 CFIN Aarhus University Hospital, Aarhus, Denmark

**Keywords:** theory of mind, mirror system, economic games, prediction error, mutual influence

## Abstract

The biological basis of complex human social interaction and communication has been illuminated through a coming together of various methods and disciplines. Among these are comparative studies of other species, studies of disorders of social cognition and developmental psychology. The use of neuroimaging and computational models has given weight to speculations about the evolution of social behaviour and culture in human societies. We highlight some networks of the social brain relevant to two-person interactions and consider the social signals between interacting partners that activate these networks. We make a case for distinguishing between signals that automatically trigger interaction and cooperation and ostensive signals that are used deliberately. We suggest that this ostensive signalling is needed for ‘closing the loop’ in two-person interactions, where the partners each know that they have the intention to communicate. The use of deliberate social signals can serve to increase reputation and trust and facilitates teaching. This is likely to be a critical factor in the steep cultural ascent of mankind.

We humans tend to think that we are the most social of all animals and our social lives the most fascinating. Surely our social nature has contributed to our success as a species. Yet, it is only recently that students of the human mind and brain have begun to explore the biological basis of our social abilities and their evolution (Adolphs 1999; Ochsner & Lieberman 2001). Of course, social psychologists have been investigating social behaviour for upwards of a century, but this work, which has contributed valuable insights on how people influence each other, occurred largely in isolation from the rest of neurobiology. Instead, the impetus for the recent marriage of social psychology with neurobiology came from comparative studies providing us with the term ‘social brain’ (Brothers 1990). This social brain, for humans at least, has a ‘theory of mind’, which enables us to predict what others are going to do on the basis of their desires and beliefs. It also has a ‘mirror system’, which enables us to understand others' goals and intentions and to empathize with their emotions by a mechanism of motor resonance. These systems are triggered by social signals, and in this paper, we will consider the nature and function of these signals in a fictitious two-way interaction with an unknown agent.

Take a typical Star Trek scenario of being stranded on an alien planet. Are there any living beings? Are they hostile or friendly? Are they like you? You need their help—and perhaps they need yours. Perhaps you can cooperate with them. Your social brain should be able to guide you to find answers to some of these questions. We start with involuntary signals and later move on to deliberate signals of communication.

## 1. Involuntary social signals

### (a) Is ‘it’ an agent?

**Figure 1.**
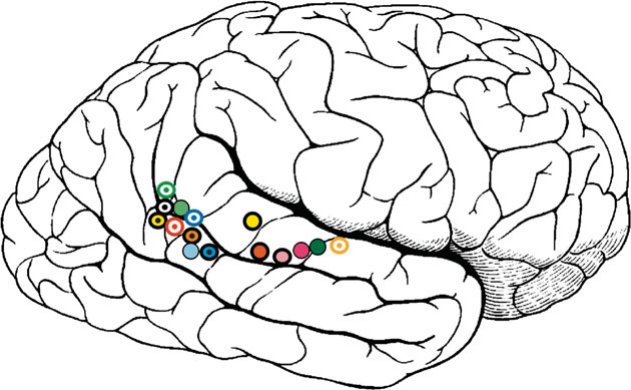
Observation of biological motion elicits activity in STS. The schematic figure shows regions where observation of many different kinds of biological motion elicits activity along STS (adapted from Allison, Puce & McCarthy, *Trends Cogn. Sci.* 2000).

Every time we move we send out involuntary signals about ourselves (this has been termed ‘public information’; Danchin *et al*. 2004). These signals inevitably tell others that we are agents. Motion dynamics seem to provide very good cues for agency. Motion cues can be isolated using point-light displays (Johansson 1973). In such displays, all information is removed except motion by showing only a few points of light located on major joints such as knees and shoulders of a person. Experiments have shown that biological motion can be picked out from other types of motion (Scholl & Tremoulet 2000). Furthermore, gender and emotion can be recognized from the movements of a point-light walker (Kozlowski & Cutting 1977; Dittrich *et al*. 1996). Biological motion of this type elicits activity in the superior temporal sulcus (STS; figure 1), especially the posterior part (pSTS). Single cells that respond to biological motion have also been identified in this brain region in the monkey (Puce & Perrett 2003). Detecting and distinguishing different kinds of biological motion is important for recognizing prey and predators as well as conspecifics. This is likely to be a very basic and universal brain mechanism, critical to survival.

### (b) What does ‘it’ have in mind?

As soon as we have established we are facing another agent, we interpret the cause of the movement. Even infants perceive moving agents as having goals and expect them to achieve these goals in a rational way, e.g. by moving along the shortest path (Csibra *et al*. 1999). When two agents act contingently, then we perceive that one caused the behaviour of the other. In our scenario, it is not necessary that the agent looks like a human. It is remarkably easy to imbue even a shapeless object with intentions as long as it appears to move in response to something you do or say (Johnson 2003). Heider & Simmel (1944) showed that geometric shapes moving in a silent animation evoked attributions of intentions in ordinary viewers. This effect is highly robust and has been investigated in neuroimaging studies in terms of intuitive attribution of mental states (Castelli *et al*. 2000). Activation of pSTS was seen, as well as of other regions relevant to theory of mind (figure 1). This suggests that perception of biological motion and the attribution of intention and other mental states share a common neural basis.

### (c) How can I know what ‘it’ will do next?

‘It’ does not look anything like us, but it moves contingently to our movements. Nevertheless, if it is like us deep down, then we can read its intentions from nothing but patterns of movements. One idea is that we do this via prediction (Kilner *et al*. 2007). Given that the object we are observing is animate and has a particular goal or intention, we can predict what movement it will make next. We then observe how well our prediction actually matches the next movement. On the basis of the prediction error we can update our reading of the goal or intention.

Evidence that pSTS is involved in such a process comes from two sources. First, pSTS activity is indeed modified by prior expectations. Wheatley *et al*. (2007) used an ingenious design in which participants were shown an object that moved in a figure-of-eight path. In one condition this object was presented as a spinning top (inanimate), while in another condition it was presented as an ice skater (animate). More activity was elicited in pSTS when this movement was perceived as representing an ice skater. Second, pSTS activity is greater when the movement does not fit with the expected intention, suggesting that this activity reflects prediction error (Pelphrey *et al*. 2003, 2004; Saxe *et al*. 2004).

Recently, Behrens *et al*. (2008) directly investigated learning via prediction error by using a task where the precise predictions of participants and, hence, prediction errors, could be estimated for every trial. The social component of this task consisted of a message from an informant who indicated to the participant, with varying degrees of accuracy, what their next response should be. A prediction error occurred when this indication turned out to be unexpectedly wrong (or unexpectedly right). Critically, these prediction errors elicited activity in pSTS. At the same time, prediction errors about the (non-social) value of an object elicited activity in the striatum, in line with findings from a number of previous experiments. Prediction error learning is an all-purpose mechanism and not specifically dedicated to social cognition. This is a useful reminder that even when the task in question is learning from other creatures, the critical computations need not be unique to social interactions.

### (d) What does ‘it’ know?

We can do even better in predicting what the alien creature will do next if we attribute and take into account its knowledge and beliefs. Knowing what other agents do not know is as important as knowing what they do know. There is now evidence (Samson *et al*. in press) that we automatically represent the knowledge of others created by their point of view. If someone else is in the room with us, and they can only see two of the four objects that we can see, the mere presence of this other person interferes with our ability to say that we can see four objects. We are slowed down when this clash of views occurs compared with when there is no such clash. This observation leads us to speculate that humans have a strong drive to fill in the gaps in other people's knowledge. There are few activities more delightful than acquiring some secret information that we can then impart to other people in confidence (Spacks 1982). In contrast, withholding information from others and deliberately deceiving them requires considerable mental effort (Vrij *et al*. 2006).

If we notice that the alien cannot see what we can see, then we have a certain advantage. We might hide something from its line of sight or else make sure we bring something into its line of sight. This kind of perspective taking does not need to be social. For example, we need to be able recognize that a place or an object is the same when we see it again from a different point of view. Tasks in which people have to infer what an object would look like from a different position (Aichhorn *et al*. 2005) elicit activity in temporo-parietal junction, a brain region closely adjacent to pSTS. This region is also activated by tasks in which it is necessary to take account of a person's out-of-date (and hence false) belief as opposed to taking account of an out-of date photograph (Saxe & Kanwisher 2003). These are both tasks that create a stark contrast of perspectives: one spatial and the other mental.

### (e) Brief excursion: the brain's theory of mind

When we interact with another person it is helpful to know something about their mental states, such as their desires, knowledge and beliefs, because this is better than anything else for predicting what they are going to do next. This vague insight was brought under experimental control by a ‘False Belief’ task developed by Wimmer & Perner (1983). For instance, we can tell where Maxi will look for his chocolate even though it has been moved to a different place when he was not there. In fact, a typical 5 year old can give you a complete explanation when given this test, and if you measure eye movements, then even infants 10–15 months old are surprised if Maxi looks in the wrong place (Onishi & Baillargeon 2005; Surian *et al*. 2007).

When we communicate with another person we also depend on an implicit and spontaneous understanding of mental states. For instance, we do not tell another person what we think they know already, and likewise they expect us to tell them something new (Grice 1989). If we follow the dark side, we can deceive and control others by taking advantage of their ignorance and making them believe things that are not true. In either case, it is important to know about the beliefs of others and to recognize that these beliefs may be different from our own and may not correspond with reality.

As yet it is not clear whether we are talking about a uniquely human ability. Premack & Woodruff (1978) first asked the question ‘Does the Chimpanzee have a theory of mind?’ It still remains controversial whether non-human primates (Povinelli & Vonk 2003; Tomasello *et al*. 2003) engage in mentalizing, as revealed, for instance, in deliberate deception. What is not controversial is that our human ability to deliberately deceive and manipulate the minds of others far outstrips that of any other creature.

However, not all humans develop this ability. Baron-Cohen *et al.* (1985) showed that children with autism have great difficulty with False Belief tasks while being able to perform other kinds of problem-solving tasks at a normal level. Even adults with autism cannot anticipate with their eye gaze where Maxi will reach to retrieve the chocolate (Senju *et al.* 2009). This in sharp contrast to normally developing children and adults.

**Figure 2.**
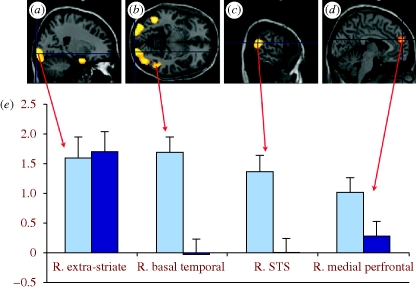
Areas of activity elicited by watching triangles whose movements evoke attributions of intentions. Activity is seen in extra-striate areas (*a* and *b*) specific to the visual nature of the stimuli as well as in (*a*) temporal pole, (*c*) STS and (*d*) medial prefrontal cortex, regions where activity has been elicited by a wide range of tasks evoking mentalizing. The diagram (*e*) contrasts activity in these areas in volunteers with Asperger's syndrome (dark blue) and controls (light blue). Asperger individuals showed less activity in areas associated with mentalizing: basal temporal, STS, medial prefrontal, but not extra-striate regions (based on data from Castelli *et al*. 2002).

Autism is defined by core deficits in social and communicative behaviour. If you observe a classically autistic child, then you can see in devastating clarity what it means not to have a spontaneous understanding of mental states. Mentalizing failure, or ‘mindblindness’, served as a highly successful explanation for the characteristic social impairments in autism. For example, it explained the inability to understand deception in the presence of intact understanding of sabotage (Sodian & Frith 1992), or the inability to understand irony with good understanding of metaphor (Happe 1993). The idea of a circumscribed mentalizing failure in autism suggested that there might be a dedicated brain system that is engaged when solving problems that require mentalizing, a prediction that was confirmed by a series of subsequent brain imaging studies (Frith & Frith 2003; Saxe *et al*. 2004). As expected, this system shows malfunction in autism as shown, for instance, in figure 2 (Castelli *et al*. 2002; see also Zilbovicius *et al*. 2006; Kana *et al*. 2009).

### (f) Is ‘it’—deep down—like me?

You may be tempted to attribute psychological states to the alien creature, but there are other checks to see if this alien feels like us. Can we tune in to each other in a way we do automatically with other humans? We tend to covertly imitate other people and feel some kind of resonance with their emotions.

### (g) ‘It’ imitates me!

When two people ‘tune in’ to each other, they tend unconsciously to imitate each other's movements and gestures and this is known as the chameleon effect (Chartrand & Bargh 1999). Furthermore, the greater the degree of imitation, the more the partners feel they have good rapport and empathy. When someone has been covertly imitated they become generally more prosocial and will give more money to charity (van Baaren *et al*. 2004). However, such effects do not occur if we become aware that we are being imitated (Lakin & Chartrand 2003). The feeling that we are part of a group, driven by unconscious motor and emotional resonance, appears to be intrinsically rewarding (Tabibnia & Lieberman 2007).

### (h) But not always

**Figure 3.**
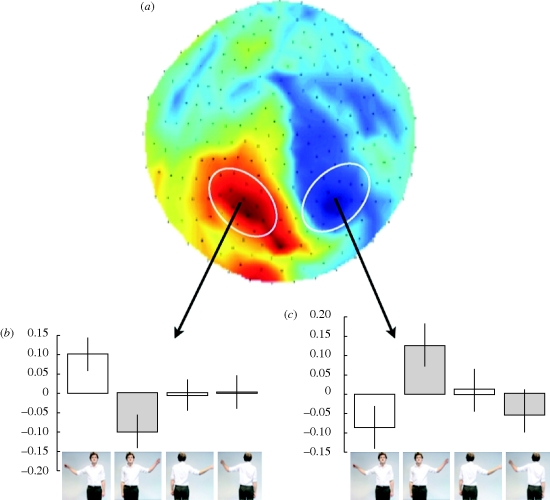
Motor resonance is modified by social interaction. Magnetoencephalography signals were measured while volunteers watched a video of an actor moving their left or right arm up and down (lower panel). Oscillations in the alpha-frequency range were relatively greater in parietal cortex contralateral to the hand being observed (middle panels), but only when the actor was facing the observer (adapted from Kilner, Marchant & Frith, *Soc. Cogn. Affect Neurosci.* 2006).

There are powerful factors that modulate motor resonance, acting through high-level systems that involve knowledge and beliefs. Less motor resonance is observed when our partner is a robot rather than a person (Kilner *et al*. 2003). This effect seems to depend more upon our belief about the nature of the agent than on the detailed behaviour of that agent (Stanley *et al*. 2007). Resonance is also modulated by the strength of the interaction (figure 3). Thus, it tends to be stronger when we have eye contact (Bavelas *et al*. 1986; Kilner *et al*. 2006).

Obviously, motor imitation is not always appropriate for successful interactions. For successful joint action the most important requirement is a common goal. To achieve this requires that most actions should be complementary rather than identical (Sebanz *et al*. 2006). Further, when pairs of subjects perform complementary tasks, each covertly represents the task requirements of the other. We can see this most strongly when the concurrent representation of another person's goal interferes with our own goal. This was shown in a joint task where two people each pressed only one button in response to a potentially incompatible aspect of the same stimulus (Sebanz 2003).

Observation and imitation of the actions of others elicit activity in inferior frontal gyrus and in inferior parietal cortex. Since these are the regions where mirror neurons have been found in monkeys, they are often identified with a human mirror system for action (Rizzolatti & Craighero 2004), which we discuss further below. Remarkably, and underlining the key role of this mechanism for successful and coordinated social interaction, when subjects are trained to perform complementary actions, even greater activity was elicited in these brain regions (Newman-Norlund *et al*. 2007).

### (i) Brief excursion: the brain's mirror system

The discovery of ‘mirror neurons’ in macaque monkeys (Rizzolatti *et al*. 1996) was a milestone in the progress of social cognitive neuroscience. These neurons, so far observed in regions corresponding to inferior frontal cortex and inferior parietal cortex, fire when the animal performs a specific action (seeing a peanut being grasped) and also when the animal observes the same specific action (grasping the peanut) being performed by someone else. The implication of these findings is that the observation of an action automatically activates the brain regions concerned with execution of that same action in the observer (Rizzolatti *et al*. 1999). Mirror neurons point to a plausible neural mechanism not only for understanding the goals and intentions of others (Gallese *et al*. 2004) but also for empathy (Decety & Myer 2008).

**Figure 4.**
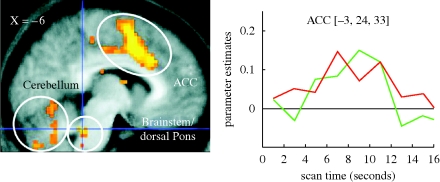
Activity is elicited in anterior cingulate cortex (ACC) by the experience of pain in the self (green line in graph on right) and by a signal indicating that a loved one is receiving pain (red line in graph on right) (adapted from Singer *et al*., *Science* 2004).

Mirror neurons have yet to be definitively identified in humans (Dinstein *et al*. 2008; but see Kilner *et al*. 2009). However, there is plenty of evidence for resonance behaviour in humans at the behavioural and the physiological level. Just think of fans at a football match who seemingly act in unison and express the same emotions. By recording from facial muscles Dimberg *et al*. (2000) showed that people automatically tend to imitate the emotional expression (frowns or smiles) seen in another face. Resonance to emotional expressions seems also to occur in non-human species (de Waal 2004). However, in humans, emotional resonance can also be elicited indirectly. The mere knowledge that someone else is currently in pain is enough to elicit activity in brain regions associated with the experience of pain (Singer *et al*. 2004) as shown in figure 4. You would be very impressed if the alien, seeing that you are injured, tried to help you.

## 2. Deliberate social signals

By now, purely through involuntary signals given out by movement, the alien in our Star Trek scenario has been revealed as a creature very much like a human. Let us assume it has a kind of social brain, but does not speak a human language. How do you both achieve a mode of communication using deliberate signals?

### (a) How can you know ‘it’ wants to communicate with you?

Let us suppose the creature is sending out a signal. How do you know it is a signal meant for you? You need to recognize that the creature wants you to attend to it. Signals that attract your attention are called ostensive. They carry with them the promise that the receiver shall gain some benefit from attending to the message (Sperber & Wilson 1995). An ostensive gesture could be visually minimal but attentionally highly conspicuous, such as the eyebrow flash (Eibl-Eibesfeldt 1972). Once a sender has initiated communication, for instance by using the eyebrow flash, then you, the receiver, will be trying to infer what the sender intends you to understand. We have speculated that anterior rostral medial prefrontal cortex (arMPFC), in which activity is elicited by many mentalizing tasks, may have a critical role in the special kind of representation that closes the loop between minds (Amodio & Frith 2006). Ostensive gestures in different modalities (eye contact and calling your name, without a message following these signals) elicit activity in arMPFC (Kampe *et al*. 2003).

### (b) ‘It’ wants to teach you

As everyone knows, it is possible to learn simply by observing others, but that is not the same as teaching. Deliberate teaching seems to be a special feature of human interactions that is not found in other primates (Maestripieri *et al*. 2002). Infants will follow the actions of adults if they are preceded by an ostensive gesture, but not otherwise (Senju & Csibra 2008). This ability is critical for learning words (Bloom 2002). First, infants can recognize when the parent is naming an object for them to learn and can distinguish this from situations in which spoken words and objects come together incidentally (Baldwin *et al*. 1996). Second, using their mentalizing ability, infants can pick out the person who knows something from the person who does not, and pay special attention to the signals coming from the one who knows (Sabbagh & Baldwin 2001). It is this ability to pick out the signals that are reliable and have communicative intent that enables infants to learn at the amazing rate of 10 new words a day (Bloom 2000). These observations suggest that humans have a special ability and perhaps even a basic desire to deliberately impart and receive knowledge from each other. Csibra & Gergely (2006) have proposed that pedagogy is a unique human ability that makes cultural accomplishments possible in the first place.

### (c) Closing the loop: ‘it reciprocates’

By now the alien will have revealed itself as a human in all but outside appearance. Can you be sure it is not a robot designed to mimic human behaviour? You can apply some more tests of its ability to communicate like a human. One particularly convincing sign of interaction is what we call ‘closing the loop’ (Frith 2007). Here is an example: we admit that by writing this paper we are attempting to influence you. But this is matched by your attempt to absorb our message and extract from it what you find useful. This is enough, but there might be consequences. You might be stung into criticizing and refuting some of our points. We, as authors, would then find out whether we explained some points badly so that you misunderstood them, or whether we ourselves had misunderstood some matters and hence misrepresented the facts. As a result of the exchange we would all have learned something we did not know before. This sort of exchange would be both a painful and satisfying example of ‘closing of the loop’. As the example also shows, mentalizing, the ability to attribute knowledge and beliefs, is heavily involved in this process.

### (d) Mechanisms of mutual influence

**Figure 5.**
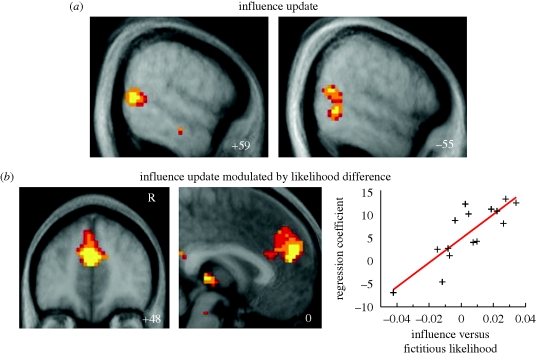
Activity is elicited when volunteers play the inspector game. The upper panel (*a*) shows activity elicited in left and right STS when the opponents' move is not what the players expect on the basis of how much influence they think they are exerting on their opponents. The lower panel (*b*) shows that there is more activity in medial prefrontal cortex in players who base their strategy on working out how much influence they have on their opponents. Yellow: *p* < 0.001; light orange: *p* < 0.05; dark orange *p* < 0.01. Adapted from Hampton, Bossaerts & O'Doherty, *Proc. Natl Acad. Sci. USA* 2008. Copyright (2008) National Academy of Sciences, USA.

A good way to approach this question is to study the behaviour of partners in competitive games in the laboratory. Here, it is not only important to predict what a partner will do next but also what a partner expects us to do next. Hampton *et al*. (2008) have developed a computational model of a strategy that allows us to represent such second-order expectations (figure 5). They call this the ‘influence’ learning model because it involves tracking the influence of one's own actions on one's opponent. They contrast this strategy with two less sophisticated strategies: one, predicting what the opponent will do next based on *the opponent's* prior actions; the other, predicting which action is most likely to win based on *one's own* past experience. In terms of their behaviour, the performance of people playing the competitive inspector game was best accounted for by the influence learning model. Hampton and his colleagues also identified brain regions where activity reflected the behaviour of the components of this model. They conclude that activity in arMPFC tracks the expected reward given the degree of influence one's past actions have on the opponent. In contrast, activity in pSTS reflects an update signal, capturing the difference between the expected degree of influence and the actual influence. This is consistent with the role of this region we have discussed previously in being more active when people's behaviour is not what we have predicted. This work could lead towards a methodology for elucidating the neural mechanisms underlying the complexities of social and strategic interactions (see also Yoshida *et al*. 2008).

### (e) You cooperate

If it looks like you are stuck for a while on your new planet, then you might do well to cooperate with the alien and join or even start a new civilization. It has been argued that the capacity for social cooperation is the lever that allowed the rapid ascent of human culture and civilization (Herrmann *et al*. 2007). We propose that this capacity relies on the explicit ability to communicate via ostensive signals based on the ability to mentalize. Mentalizing ability may well have been a major stimulus for the development of spoken as well as written language and their use in teaching. You might start a writing system based on social signals. If you wish to use the alphabet, then speech sounds are key. But whatever you use, a written system of communication allows knowledge to be conveyed from one agent to another without them ever meeting each other, thus separating communication from the very stimuli that normally drive it. In this way, previous generations can influence later generations far into the future.

But even immortalized communication is not enough to explain cultural evolution. We can also count on altruistic cooperation (Moll & Tomasello 2007) with the spectacular results produced by stable social structures, such as cities, markets, temples, courts of law, prisons, universities, satellite transmission and the Internet. Plentiful examples exist in science fiction to imagine this proliferation even on your new planet. Now, in human societies, we know that cooperation is not entirely free of self-interest. We suggest that one force that drives us to cooperate is the wish to build a good reputation. A good reputation is of immense value in social interactions. This is illustrated vividly by the so-called audience effect: you behave differently, more empathically, more generously, more honestly, if you are observed by others (Hoffman *et al*. 1996).

### (f) You build your reputation

Smith (1759) in his ‘Theory of Moral Sentiments’ suggested that underlying the drive to acquire wealth is a more fundamental desire to acquire a good reputation. ‘The rich man glories in his riches, because he feels that they naturally draw upon him the attention of the world’ while for the poor man, in contrast, ‘to feel that we are taken no notice of, necessarily damps the most agreeable hope, and disappoints the most ardent desire, of human nature’ (TMS, I,III,16). Furthermore, he suggests that ‘Men have voluntarily thrown away life to acquire after death a renown which they could no longer enjoy’ (TMS, III,I,12). The implication here is that the desire to be noticed and to have a good reputation cannot only create a drive to acquire wealth, but also a drive to behave altruistically.

We believe that ostensive signalling is crucial in building a reputation. Some anecdotal evidence from real-life trading suggests that this may be so. A new form of ostensive signalling appears to have evolved in the move from face-to-face trading pits to anonymous electronic markets. Market trading depends heavily upon trust, and trust requires that you know who you are trading with. When face-to-face trading was replaced by numbers on a computer screen such identity was no longer supplied. However, in some supposedly anonymous electronic markets, participants sometimes signal their identities by offering to buy not 10 000 000 shares, but 10 000 467, or bidding at $92 700 059. Here, the ‘467’ and ‘59’ at the end of the big numbers act like a codename (Zaloom 2006). It obviously makes no economic sense to bid $92 700 059 rather than $92 700 000. This is the sign of a deliberate signal indicating that this is ‘$59 trader’ bidding.

### (g) You show trust

Deliberate signalling in which both sender and receiver know that signals are being exchanged is a prime example of ‘closing the loop’. You need to know that the alien knows that you are signalling. Further, you want the alien to believe that you know that it trusts you. This ‘common knowledge’ is important for maintaining cooperation in interactive trust games, such as the Ultimatum game and the Prisoner's Dilemma. If mutual trust breaks down, then cooperation ceases and both parties suffer through earning less reward. A real problem for social interactions is how to get back to a state of cooperation once mutual trust has been lost. We suggest that, here too, deliberate signalling has a critical role.

### (h) You forgive

In your tentative interactions with the alien, a breakdown has occurred. But your attempts at cementing a cooperative interaction will not necessarily be stopped by this. It appears that evolution has biased humans to behave in a prosocial and cooperative manner. This seems to be our default mode of behaviour when we are not thinking very deeply about what we are doing (Frith & Frith 2008*a*). Many economic games can be played perfectly well at this level. A simple strategy of tit-for-tat (strict reciprocity: cooperate if your partner cooperates, defect if he defects) will usually give the best results (Axelrod & Hamilton 1981). But what happens when things go wrong?

Van Lange *et al*. (2002) examined what happened when noise was introduced into a trust game. In the game you are asked to invest money in your partner, but the amount you invest is randomly altered. As a result, your partner might receive a smaller than expected investment. This could easily lead to a breakdown in trust if your partner blames you rather than the system and returns with an even smaller investment. Indeed, in the experiments the simple tit-for-tat strategy no longer maintained cooperation. However, cooperation could be maintained if a partner behaved somewhat more cooperatively than the actor did in the previous interaction (i.e. tit-for-tat plus one). Forgiving behaviour was also observed by King-Casas *et al*. (2008) in a study where the breakdown of cooperation was caused by the abnormal behaviour of participants with borderline personality disorder, playing with healthy partners. When this breakdown occurred the healthy partners engaged in ‘coaxing’ behaviour. This was defined as giving back more than had been offered (i.e. a third or more of the tripled investment) even though the offer was low and is similar to Van Lange's tit-for-tat plus one. The participants with borderline personality disorder neither indulged in nor responded to coaxing behaviour. One of the more far-flung promises of social cognitive neuroscience is that studies of this type could have application in the resolution of social conflict.

## 3. Beyond star trek

We have tried to make a case for the central importance of social signals and have given examples of how even complex social interactions between two partners can be brought under experimental control in the laboratory. How can we use the knowledge gained so far to improve our often disastrous social relations in real life? One optimistic example is the effect of coaxing behaviour in economic games. If this is the effect of deliberate signalling, then we speculate that such paradigms can become tools to probe potential sources of misunderstanding. For example, you can sense when a prediction error has occurred by monitoring not simply *what* is being said, but *why* in this form rather than another, and you do this automatically as you engage in ostensive communication (Sperber & Wilson 1995). If my partner in trading pays me back more than I have invested in him, this is not rational. So, I need another interpretation of his behaviour. I infer that this is more than an economic exchange. Rather it is a deliberate signal asking me to trust him, as he trusts me.

How can impairments in social skills, common in many psychiatric disorders, be remedied by therapy? For instance, people with autism, who have problems with spontaneous theory of mind (Frith 1989, Senju *et al.* 2009), should show an absence of regard for their own reputation, in sharp contrast to their ability to judge others as being fair or unfair, mean or generous. We typically show that we have regard for reputation by the difference in our attitude when we interact with other people as compared with interacting with a computer. Thus, we might expect people with autism to make no distinction between computers and people when playing interactive games. Preliminary evidence that this is the case comes from the study by Chiu *et al*. (2008; see comment by Frith & Frith 2008*b*). If this is confirmed, we doubt that it is wise to focus on improving social skills via robot interactions, notwithstanding the fact that some therapists keenly advocate such methods. Instead, we look forward to seeing results from learning paradigms, which investigate the failure to respond to, and get rewards from social stimuli, and those that test the speculative hypothesis that people with autism learn less well from prediction errors about social stimuli. If this were the case, it might be possible to teach by eliciting very large prediction errors and decreasing them very gradually. This is quite the opposite of the current ideal, which tends to rely on the teacher behaving in a highly predictable manner.

The young field of social cognitive neuroscience faces many pressing questions. We know very little about the causes of individual differences in social abilities and their genetic basis. Can social drugs such as oxytocin be used to improve social abilities in autism (Ebstein *et al*. 2009)? Can they be used to enhance social ability even in otherwise healthy people? How do social cognitive abilities develop in relation to brain maturation? Social abilities are in evidence at an amazingly young age. Even newborns orient to faces and voices rather than any other stimuli. They soak up information from other human beings by following their gaze and by responding to deliberate signals of communication. However, we urgently need to know more about later social development. Fortunately, adolescence is now being studied as a phase of brain reorganization concurrent with major changes in social interests and skills (Blakemore 2008).

Readers will have noticed that we are inveterate enthusiasts and would find it difficult to be sceptical about the future of social cognitive neuroscience. Of course, we realize that methodological breakthroughs are needed to reveal the relevant physiological processes in the brain and to link them meaningfully to mind and behaviour, and we strongly believe that, to flourish, social cognitive neuroscience must remain in touch with general cognitive neuroscience. Many would agree that the most challenging frontier for the biological sciences now is to understand how the human brain produces the mind. If it can be argued that the brain has evolved to enable us to interact and communicate with each other, then finding the basis of this ability will be the key. This is why we would love to communicate with creatures on Mars.
